# Un sacré sacrum!

**DOI:** 10.11604/pamj.2018.30.260.13452

**Published:** 2018-08-07

**Authors:** Dhia Kaffel, Amal Ben Ouhiba, Wafa Hamdi, Kaouther Maatallah, Hend Riahi, Mouna Chelly Bouaziz, Mohamed Fethi Ladab, Mohamed Montacer Kchir

**Affiliations:** 1 Service de Rhumatologie, Institut Mohamed Kassab, Mannouba, Tunisie; 2 Service de Radiologie, Institut Mohamed Kassab, Mannouba, Tunisie

**Keywords:** Ostéolyse post-fracturaire, sacrum, pseudotumeur, Post-fracture osteolysis, sacrum, pseudotumor

## Abstract

L'ostéolyse post-fracturaire du sacrum est une entité radiologique rare mimant une pathologie grave. Nous en rapportons une observation. Notre patiente a été perdue de vue après que l'indication de biopsie soit retenue, et elle n'a reconsulté qu'après 4 mois. Au moment de la réalisation des coupes de repérage précédant la biopsie, nous avons constaté un changement d'aspect de la lésion associant une image linéaire (correspondant au trait de fracture) à une condensation des berges. L'évolution était bonne, sous repos, après 6 mois de recul. Notre cas illustre l'aspect atypique de l'imagerie et les difficultés diagnostiques qui s'y associent. La particularité de notre patiente est que sa symptomatologie était masquée par un conflit disco-radiculaire avec la racine L5 droite et que l'ostéolyse sacrée était de découverte fortuite à la tomodensitométrie.

## Abstract (en)

Post-fracture osteolysis of the sacrum is a rare radiological entity mimicking a serious disease. We report the case of a female patient lost to follow-up after biopsy indication. She presented again after 4 months. During multi-stage bone registration prior to the biopsy, we noticed a change in the appearance of the lesion, associating a linear image (corresponding to the fracture line) with condensation at the edges. Patient’s evolution was good after a period of rest at 6-months follow-up. This study highlights the atypical imaging appearance and the diagnostic difficulties associated with post-fracture osteolysis of the sacrum. The particularity of our patient is that symptoms were masked by disc-related nerve root impingement with right L5 root and that the sacrum osteolysis was unexpectedly discovered on CT scan.

## Introduction

L'ostéolyse post-fracturaire du sacrum est une entité radiologique rare mimant une pathologie infectieuse ou tumorale. Elle est secondaire à un mode évolutif inhabituel d'une fracture sacrée et implique souvent des explorations lourdes et invasives. Nous en rapportons une observation qui illustre l'aspect atypique de l'imagerie et les difficultés diagnostiques qui s'y associent.

## Patient et observation

Une femme âgée de 53ans, sans antécédents pathologiques notables (notamment pas de traumatisme du bassin), a consulté pour une lombosciatique L5 droite d'horaire mécanique résistante à un traitement symptomatique bien conduit. La radiographie du rachis avait noté une petit pincement L4-L5 avec une arthrose interapophysaire postérieure. La tomodensitométrie du rachis lombaire et du bassin avait retrouvé une lombodiscarthrose étagée associée à une discopathie protrusive L4-L5 conflictuelle avec la racine L5 droite expliquant la symptomatologie; cependant, elle a aussi montré une importante lésion ostéolytique de l'aileron sacré droit d'allure suspecte de 45mm de grand axe. L'IRM avait montré une lésion osseuse de l'aileron sacré droit comblant le trou sacré S1 droit, contenant une zone de nécrose et associée à une importante réaction des parties molles en regard. Il n'existait pas de syndrome inflammatoire biologique. Le bilan phosphocalcique sanguin était sans anomalies. L'IDR à la tuberculine était négative. Les sérologies de Wright et de Widal étaient négatives. La radiographie du thorax, l'échographie abdomino-pelvienne, l'échographie cervicale ainsi que l'écho-mammographie étaient sans anomalies. Une biopsie osseuse scanno-guidée a été indiquée, cependant la patiente a été perdue de vue et n'a reconsulté qu'après 4 mois. Au moment de la réalisation des nouvelles coupes scannographiques de repérage précédant la biopsie on a constaté un changement d'aspect de la lésion associant une condensation des berges ainsi qu'une image linéaire correspondant au trait de fracture sacré droit. La patiente n´a donc finalement pas eu la biopsie programmée initialement et elle a été confiée aux orthopédistes avec une bonne évolution, sous repos, après 6 mois de recul. La mesure densité minérale osseuse était par ailleurs normale.

## Discussion

Les ostéolyses post-fracturaires du bassin, sont décrites surtout au cours des fractures de contrainte par insuffisance osseuse. Le terrain sur lequel surviennent ces fractures est souvent le même impliquant une femme âgée de plus de 50 ans avec ostéoporose. Néanmoins d'autres facteurs favorisants ont été mis en évidence: radiothérapie pelvienne [1,2], polyarthrite rhumatoïde [3,4], hyperparathyroïdie [5], alcoolisme [5,6], corticothérapie [2,7], ostéomalacie [2,7] et antécédent de prothèse de hanche [4]. La fracture survient soit après un traumatisme tel qu'une chute [8,9] ou un traumatisme plus minime, soit un excès d'exercice physique [10], ou, plus rarement comme dans notre observation, sans facteur déclenchant [3]. La symptomatologie la plus fréquente des fractures du sacrum est représentée par des douleurs du sacrum d'horaire mécanique, exacerbées par l'effort, rendant la station debout et la marche difficiles [2,7].

La particularité de notre cas est que sa symptomatologie était masqué par le conflit disco-radiculaire. Initialement, Les fractures peuvent passer inaperçues sur les radiographies standard du fait de leur discrétion [8,9], ce qui pourrait retarder le diagnostic de quelques semaines ou mois. Le délai moyen de guérison est de 3 mois [11]. La scintigraphie est un examen très sensible [4]. Elle montre précocement une hyperfixation localisée au niveau du sacrum. Elle cartographie aussi les autres foyers fracturaires [11]. Habituellement et contrairement à notre cas, la tomodensitométrie montre plus précocement le trait de fracture et fait la distinction entre une ostéolyse maligne et bénigne [1,12]. L'IRM peut s'avérer utile dans les fractures du sacrum afin d'éliminer une lésion métastatique associée [2,7]. Classiquement, elle donne un aspect en « H ».

Cependant, dans les rares cas d'ostéolyse post-fracturaire, elle peut faussement montrer une ostéolyse pseudo maligne associée à un œdème osseux et des parties molles en regard. Dans les cas douteux, la biopsie peut rectifier le diagnostic. Néanmoins elle devrait être évitée car la lecture peut être parfois difficile montrant un tissu nécrotique, un foyer de fracture en organisation fibreuse [1,13] et des diagnostics erronés comme un ostéosarcome ou chondrosarcome peuvent être évoqués à tort [1,13]. Le mécanisme le plus souvent invoqué pour expliquer de telles ostéolyses est biomécanique [13]: d'un côté, l'existence d'une fracture du sacrum qui favorise, par rupture de l'anneau pelvien, les fractures des branches pubiennes et le déséquilibre du basin; de l'autre une mobilité anormale du foyer de fracture favorisée par les mouvements de cisaillement à la marche. La conduite thérapeutique préconisée est le repos et les antalgiques [14]. Certains auteurs préconisent une cimentoplastie. Enfin, une guérison a semblé plus rapide (bien que de façon non significative), suite à l'utilisation d'œstrogènes, de calcium et de bisphosphonates (pamidronate) par d'autres auteurs [15].

## Conclusion

L'ostéolyse post-fracturaire est une pathologie bénigne, rare et qui doit être évoqué devant toute lésion lytique du sacrum.

## Conflits d’intérêts

Les auteurs ne déclarent aucun conflit d'intérêts.
